# Supplementary material for: Switchable plasmonic routers controlled by external magnetic fields by using magneto-plasmonic waveguides
Source: Sci Rep. 2018 Jul 12;8:10584. doi: 10.1038/s41598-018-28567-8 (PMC6043596; doi:10.1038/s41598-018-28567-8)
Supplement: Supplementary file 1 — Supplementary Information [file 41598_2018_28567_MOESM1_ESM.pdf]

Switchable plasmonic routers controlled by external magnetic fields by using magneto-plasmonic waveguides

Kum-Song Ho[1], Song-Jin Im[1,*], Ji-Song Pae[1], Chol-Song Ri[1], Yong-Ha Han[1], and J. Herrmann[2,+]

[1]Department of Physics, Kim Il Sung University, Pyongyang, Democratic People's Republic of Korea
[2]Max-Born-Institute for Nonlinear Optics and Short Pulse Spectroscopy, Max-Born-Str. 2a, D-12489 Berlin, Germany
[*]ryongnam31@yahoo.com
[+]jherrman@mbi-berlin.de

ABSTRACT

Supplementary information

Derivation of Eq. (4)

Let's remind the dispersion relation Eq. (3).

$$e^{-2k_m t} = \frac{q_m + q_{-g}}{q_m - q_{-g}} \frac{q_m + q_g}{q_m - q_g}, \tag{0.1}$$

where $q_m = k_m/\varepsilon_m$, $q_g = (\varepsilon_d k_d + g\beta) / \left(\varepsilon_d^2 - g^2\right)$, ${k_m}^2 = \beta^2 - k_0^2\varepsilon_m$, $k_d^2 = \beta^2 - k_0^2(\varepsilon_d^2 - g^2)/\varepsilon_d$, t: thickness of the metal film, β: propagation constant , ε_m and ε_d: permittivity of the metal and the ferromagnetic dielectric, respectively.

For a metal film thickness larger than the skin depth $\delta = 1/2k_m$, in first approximation of $|\Delta\beta/\beta_0|$, following equations can be obtained.

$$e^{-t/\delta} = \frac{(q_m + q_d)^2 - \left(g\beta_0/\varepsilon_d^2\right)^2}{(q_m - q_d)^2 - \left(g\beta_0/\varepsilon_d^2\right)^2}, \tag{0.2}$$

$$\begin{aligned} q_m &= -q_0\left(1 + \frac{\beta_0\Delta\beta}{q_0^2\varepsilon_m^2}\right), \\ q_d &= q_0\left(1 + \frac{\beta_0\Delta\beta}{q_0^2\varepsilon_d^2}\right), \end{aligned} \tag{0.3}$$

where $q_d = k_d/\varepsilon_d$, $\beta = \beta_0 + \Delta\beta$, $\beta_0 = k_0[\varepsilon_d\varepsilon_m/(\varepsilon_d + \varepsilon_m)]^{1/2}$, $q_0 = k_0(-\varepsilon_d - \varepsilon_m)^{-1/2}$.

By substituting Eq. (0.3) to Eq. (0.2), we can get the following analytical equation.

$$\Delta\beta^2 = \beta_0^2\left[\frac{4(\varepsilon_d/\varepsilon_m)^2 e^{-t/\delta}}{\left(1 - \varepsilon_d^2/\varepsilon_m^2\right)^2} + \frac{g^2}{\left(1 - \varepsilon_d^2/\varepsilon_m^2\right)^2(-\varepsilon_m\varepsilon_d)}\right]. \tag{0.4}$$

From Eq. (0.4), we obtain Eq. (4).

$$\begin{gathered} \beta^{e(o)} = \beta_0 + (-)\sqrt{(\Delta\beta_0)^2 + (\Delta\beta_g)^2}, \\ \Delta\beta_0 = \frac{2(-\varepsilon_d/\varepsilon_m)\exp(-t/2\delta)}{1 - \varepsilon_d^2/\varepsilon_m^2}\beta_0, \qquad \Delta\beta_g = \frac{g}{\left(1 - \varepsilon_d^2/\varepsilon_m^2\right)\sqrt{-\varepsilon_m\varepsilon_d}}\beta_0, \end{gathered} \tag{0.5}$$

where the superscripts o and e represent the odd and the even modes, respectively.
